# Characteristics and Trends of NIH-Funded Opioid Use Disorder Clinical Trials During the Opioid Epidemic With a Focus on Gender

**DOI:** 10.7759/cureus.82227

**Published:** 2025-04-14

**Authors:** Jyothika Yermal, Gabriel P Costa, Jeremy Weleff, Wilhemina Koomson, Brian S Barnett, Akhil Anand

**Affiliations:** 1 School of Medicine, Case Western Reserve University School of Medicine, Cleveland, USA; 2 School of Medicine, University of Ribeirão Preto, Ribeirão Preto, BRA; 3 Department of Psychiatry, Yale School of Medicine, New Haven, USA; 4 School of Medicine, Cleveland Clinic Lerner College of Medicine, Case Western Reserve University, Cleveland, USA; 5 Department of Psychiatry and Psychology, Cleveland Clinic, Cleveland, USA

**Keywords:** addiction, clinical research, equity, funding, nih grants, opioid epidemic

## Abstract

Objectives

This study aims to analyze all funding awarded by the National Institute of Health (NIH) for opioid use disorder (OUD) clinical trials during the opioid epidemic and investigate whether there exist a difference in funding based on gender of principal investigators (PIs) and the wave of the opioid epidemic during which the trial began.

Methods

NIH-funded clinical trials related to OUD during the opioid epidemic between March 1, 1997, and February 28, 2023, were extracted from ClinicalTrials.gov and NIH RePORTER. Data extracted from each project included grant type, grant category, grant funding amount, and the PI's affiliation, academic rank, highest degrees, and gender. Non-parametric statistical analysis was performed using Kruskal-Wallis testing to investigate for discrepancies in NIH funding between genders and waves of the opioid epidemic.

Results

A total of 197 trials were identified as funded during the opioid epidemic, for a total of $1,646,512,558. Of the grants, 85.8% (N=169) were awarded during the third wave of the opioid epidemic; they received a median of $2,482,291 per year, significantly more than the second wave (p=0.0045). Male PIs (54.3%, N=107) received the highest number of grants, but they did not receive significantly more funding compared to female PIs (p=0.8646). A statistically significant difference in funding between the waves (p=0.0015) was found, with projects starting during the third wave receiving more average yearly funding (N=169) than the first two waves (N=28).

Conclusion

NIH funding amount and number of projects funded increased throughout the third wave of the opioid epidemic, potentially due to increased NIH initiatives during this time. Unlike other documented trends outlining disparities in funding between male and female researchers in academic medicine, there was no statistical difference in funding for clinical trials during the opioid epidemic. Further analysis is needed to ensure a diverse research landscape and determine areas for future improvement.

## Introduction

Opioids are the leading cause of drug-related fatal overdoses in the United States [1]. From 2010 to 2021, opioid-related fatal overdoses increased almost fourfold, resulting in 109,411 deaths in 2022 [2]. The opioid epidemic is often described in "waves." The first wave started in the mid-1990s, largely attributed to the introduction of OxyContin to the market in 1996 and the excessive prescribing of opioids driven by the aggressive promotion of "pain as the fifth vital sign" by the American Pain Society and pharmaceutical companies. The second wave started in 2010 when new opioid prescribing guidelines emerged. Federal regulations on opioid prescribing became stricter and more punitive, leading to an abrupt and significant reduction in prescribing nationwide, which caused opioid-dependent patients to turn to illicit opioids, such as heroin [3]. The third wave started in 2013 with the rise of high-potency synthetic opioids like fentanyl. Unfortunately, the current third wave shows no indications of slowing down and has intensified following the pandemic [4]. Experts argue that we are now experiencing a fourth wave of the opioid crisis, which involves the co-occurring use of psychoactive stimulants [5].

To address the present opioid epidemic, it is crucial to support research on opioid use disorder (OUD). Funding from the National Institutes of Health (NIH) plays a vital role in advancing this research [6]. Every year, the NIH and its subsidiary bodies award over 50,000 grants worth $32 billion to more than 300,000 researchers [7]. Being awarded an NIH grant is a highly competitive and career-defining achievement, as it supports their laboratories and enhances their professional opportunities [8,9]. Clinical researchers thus must understand NIH grant allocations, awarded grant types, and funding trends to help in the development of their grant applications.

Although there have been almost equal percentages of women and men graduating from medical school since 2005 [10], women remain underrepresented in academic medicine [11], as they hold fewer full professorial positions, earn lower salaries, and receive fewer research citations than their male counterparts [12]. There is a rising concern that NIH funding distribution is not always equitable across genders [13]. Diversity in research adds different experiences and perspectives, enhancing the ability to solve complex problems and generate higher-quality research [14,15]. In the context of clinical research, it can also ensure that participants and, consequently, the study's findings are representative of minorities and accurately reflect the broader population [16].

Currently, there is limited research examining gender diversity among principal investigators (PIs) who secure clinical research funding in addiction medicine. The aim of this study is to analyze NIH funding for OUD clinical trials across the three waves of the opioid epidemic and investigate if there were any disparities in funding based on the gender of the PI. Additionally, we examined whether there were any differences in funding based on the PI's highest academic degree, academic ranking, and the grant category itself. We hypothesized that there would be disparities in funding across these variables and that the funding landscape would undergo significant changes with each wave of the opioid epidemic.

## Materials and methods

Data source

We used the ClinicalTrials.gov trial registry to identify all completed and ongoing interventional clinical trials for OUD that were posted by February 28, 2023. We then cross-verified all trials and gathered additional grant and financial information on each trial using NIH RePORTER, as it was not possible for us to filter for clinical trial grants on RePORTER. PIs' demographic details were gathered from publicly available profiles on institutional websites using the listed PI affiliation and name. Lastly, the data was cataloged into the three waves based on the Centers for Disease Control and Prevention's timeline of the opioid epidemic: first wave: January 1, 1996-December 31, 2009; second wave: January 1, 2010-December 31, 2013; and third wave: January 1, 2013-present [17]. This study did not require institutional review board approval as the data was publicly available.

Study selection and data extraction

Data were collected on March 1, 2023, by two authors (JY and WK). In our query, we included the following conditions: Opioid-Use Disorder; Opioid-Related Disorders; Opioid Misuse; Opioid Dependence; Analgesics, Opioid; Opioid Use; Maternal Opioid Use Disorder; Opiate Addiction; Addiction, Opioid; Opioid Abuse; and Substance Use Disorders. The resulting data was then refined by excluding non-interventional clinical trials that did not directly investigate OUD, as well as any studies that did not claim to be funded by the NIH. The following data were collected from each research project: (i) grant title, (ii) grant type, (iii) grant category, (iv) amount of funding per grant, (v) the year funding began, (vi) the year funding ended, (vii) primary PI's full name, (viii) primary PI's affiliation, and (ix) primary PI's gender. Gender was determined based on the pronouns used in the PIs' profiles, with PIs referenced as he/him listed as men and those referenced as she/her as women. PIs who received grants for more than one distinct project were classified as "multi-grant" awardees. If any data were missing, two authors (JY and WK) attempted to contact project contacts, and studies that did not have all the necessary data were excluded. Discrepancies were resolved by consensus among three authors (JY, WK, and AA).

Funding values

Total funding was determined using the fiscal year total cost, which encompasses all funding (direct and indirect costs) of a grant from October 1st to September 30th of the following year and is reported on NIH RePORTER [18]. For projects that lasted multiple years, total funding was calculated by summing the total costs for each year. The average yearly funding was calculated by dividing the total funding by the number of years that the project received funding.

Statistical analysis

Analysis was conducted for both total funding and average yearly funding. Differences in funding distribution across various groups, such as opioid epidemic waves, gender, degrees, ranks, and grant categories, were tested using the Mann-Whitney U and Kruskal-Wallis tests. This approach was used as the Shapiro-Wilk test results indicated that the funding data was not normally distributed. Following a significant Kruskal-Wallis test, we performed Dunn's post hoc tests with Bonferroni correction to identify which specific pairs of groups differed. P-values of less than 0.05 were considered significant. R software version 4.2.3 was used for statistical calculations [19].

## Results

A total of 229 projects were initially identified using the ClinicalTrials.gov trial registry. Thirty-two projects were excluded because they could not be verified in NIH RePORTER. Two additional projects were excluded because of missing principal investigator (PI) information. In the end, we analyzed 197 OUD clinical trials. Due to the paucity of trials awarded during the first and second waves, PI demographics and grant types could not be compared between waves. Table 1 and Table 2 provide the funding values for each wave and gender.

**Table 1:** NIH funding by gender NIH: National Institute of Health, USD: United States dollar, IQR: interquartile range

Gender	Sum of total funding (USD)	Median of average yearly funding (USD/year)	25th percentile (USD)	75th percentile (USD)	IQR (USD)
Women	$706,325,695	$2,120,169.00	$768,413.00	$5,119,789.25	$4,351,376.25
Men	$940,186,863	$2,545,657.50	$746,689.50	$5,124,549.50	$4,377,860.00

**Table 2:** NIH funding by waves of the opioid epidemic NIH: National Institute of Health, USD: United States dollar, IQR: interquartile range

Epidemic wave	Sum of total funding (USD)	Median of average yearly funding (USD/year)	25^th^ percentile (USD)	75th percentile (USD)	IQR (USD)
First wave	$140,494,600	$2,299,220.00	$1,297,103.00	$3,940,772.50	$2,643,669.50
Second wave	$22,898,445	$806,796.00	$554,348.00	$2,668,008.00	$2,113,660.00
Third wave	$1,483,119,513	$2,482,290.50	$768,413.00	$5,479,593.25	$4,711,180.25

Funding through the opioid epidemic

A total of $1,646,512,558 NIH grant funding was identified for OUD clinical trials. Fifteen projects were awarded grants during the first wave of the epidemic; a total of $140,494,600 (8.5% of the total throughout all waves) with a median of $2,299,220/year (IQR: $2,643,669.5) was awarded. Thirteen trials were awarded during the second wave; a total of $22,898,445 (1.4% of the total throughout all waves) with a median of $806,796/year (IQR: $2,113,660) was awarded. One-hundred sixty-nine trials were awarded during the third wave; a total of $1,483,119,513 (90.1% of the total throughout all waves) with a median of $2,481,291/year (IQR: $4,711,180.25) was awarded.

**Figure 1:**
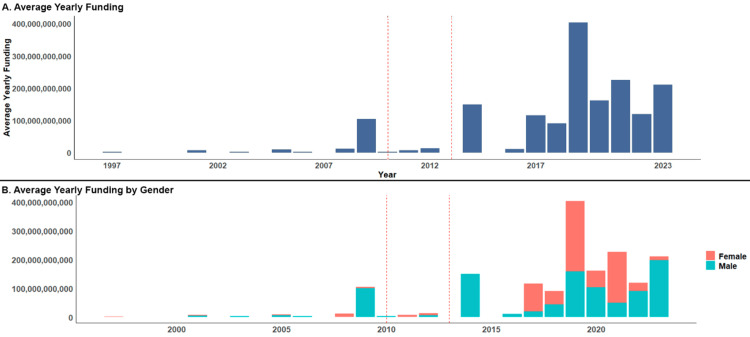
Average yearly NIH funding allocations for opioid clinical projects from 1997 to 2023 Dashed red lines denote separate waves of the opioid epidemic. NIH: National Institute of Health

A statistically significant difference in average yearly funding was found between all three waves (p=0.002). Subsequent Dunn's post hoc tests revealed a significant difference in funding between the second and third waves (p=0.005). Figure 1A illustrates the funding trends throughout the epidemic.

Funding by gender

Out of a total of 197 grants, 90 (45.7%) were awarded to women and 107 (54.3%) were awarded to men. Among the PIs who received multiple NIH grants, 32 were women (44.4%) and 40 were men (55.6%). Throughout the epidemic, women received a total of $708,000,000, while men received a total of $945,000,000. Women received a median of $2,120,169/year (IQR: $4,351,376.25) per grant, while men received a median of $2,545,657.50/year (IQR: $4,377,860) per grant. Despite the observed differences in the number of grants awarded, the Mann-Whitney U test found no significant difference in funding between male and female PIs (p=0.865). Figure 1B illustrates funding by gender.

## Discussion

We examined the gender distribution of PIs who received funding from the NIH for clinical research for OUD and tracked funding allocation through the opioid epidemic. Within the confines of this dataset, there was no correlation between the gender of the PIs and varying funding outcomes. However, a statistically significant disparity in the average yearly funding was observed across the epidemic waves, with a 308% increase noted in the third wave compared to the second wave.

In the third wave, there was a significant increase in the number of grants awarded funding compared to the previous two waves. The average yearly funding also significantly increased compared to the second wave. This rise in awards and average yearly funding could be attributed to newly established initiatives by the NIH aimed at addressing the opioid epidemic. For instance, the Helping to End Addiction Long-term Initiative began funding OUD projects in 2018 and has awarded more than $2.5 billion between 2019 and 2022 [20]. This initiative led to several subsidiary programs, including the Justice Community Opioid Innovation Network, which aims to improve care for those in the justice system, and the HEALing Communities Study, which investigates treatment and harm reduction methods in local communities. The National Drug Abuse Treatment Clinical Trials Network has also been expanded and is currently working to increase the number of clinical trials conducted nationwide [21]. Other factors contributing to the increase in NIH funding include government initiatives such as the 2016 Government Addiction and Recovery Act and the formation of the President's Commission on Combating Drug Addiction and the Opioid Crisis [22]. Notably, there was a decrease in grant funding in 2020 because of the COVID-19 pandemic. However, as OUD and overdoses continue to increase in the wake of the pandemic, we anticipate that funding for this research will continue to increase as it did prior to the pandemic [23].

Although fewer female PIs received NIH-funded grants compared to male PIs, there were no significant differences in median funding between genders. This result was unexpected, as previous studies have demonstrated that female PIs in other medical fields face disadvantages in both the number and size of grants they receive [24-27]. This unexpected finding could be attributed to a smaller gender gap in psychiatry [28] and potential gender parity in the peer review process for addiction research, which could increase the participation of female PIs in this subspecialty [29].

Another explanation could be the deliberate steps taken by the NIH to boost the representation of female NIH grant awardees in medicine since 2018 [30]. This has involved policy changes and thus the establishment of dedicated offices [31] and the provision of funding opportunities through initiatives such as the Diversity Supplement Program [32] and the Next Generation Researchers Initiative [33]. These efforts aim to enhance backing for early career researchers and other underrepresented groups, including women. The National Institute on Drug Abuse also launched the Diversity Scholars Program, which aims to promote the funding success of early career investigators from diverse backgrounds [34]. However, it is still too early to determine whether these programs have had a significant impact. In addiction medicine, the combination of gender parity in the peer review process, NIH support for traditionally underrepresented groups, and a more balanced pool of applicants results in a lack of such stark inequity.

The NIH is working toward a more equitable grant process [35]. Thus, we expect that there will likely be an increase in the number of female PIs receiving awards in OUD research. Since we do not know the makeup of the applicant pool for certain, we cannot speculate whether the proportion of women compared to men will change. It is plausible to expect that, over time, grant funding distribution will align with the applicant pool's demographics. However, further research is necessary to confirm whether this trend applies universally across all medical research.

Limitations

This study has several limitations. While the Clinical Trials and NIH RePORTER websites were thoroughly cross-referenced, studies may have been missed if they received grant funding under a different title, PI, institution, and location. Although we followed previously cited protocols [24-26], there may be inaccuracies due to information being taken from public websites. Primary PI affiliation was used to find biographies of PIs, which were then analyzed for pronoun use. The gender identities of the researchers may have changed since the time they were awarded the grant. Their identities also may not have truly aligned with the pronouns used in these profiles; although we did not find any pronouns other than he/him and she/her being used, there may have been individuals that identified with genders other than those recorded. We also did not have access to other parameters, such as race/ethnicity, medical specialty or PhD focus of PIs, or the prestige of their education and affiliation. These factors may have played a significant role in grant funding opportunities and confounded the relationship between gender and funding [36-38]. We were unable to compare PI demographics between the waves due to the scarcity of studies in the first and second waves. PIs listed as single grant awardees in our study may have received prior NIH funding in a different field that was not captured in our specific dataset. We organized projects by their start date, which precluded any analysis to capture how much funding was being spent by the NIH each year. Importantly, we were unable to report on studies that did not receive grant funding, which would have added more context and allowed us to compare the success rates between demographics, grant types, and waves of the epidemic.

## Conclusions

This study highlights a positive shift in funding for OUD clinical trials in the third wave of the opioid epidemic, as there were dramatic increases in total funding, average yearly funding, and the number of NIH grants awarded during the last wave of the opioid epidemic. Importantly, there were no statistically significant differences in total funding for PIs of different genders, indicating that there may be no gender bias in funding within this field of research. However, there may be other demographic factors that are more contributory than gender, as these trends have been observed in other fields of NIH research. As the opioid epidemic continues, it is crucial to continue analyzing the types of studies funded by NIH and the characteristics of the PIs who receive these grants to ensure that this necessary field of research is being funded equitably.
